# Evaluation and Treatment Options for Abnormal Uterine Bleeding in Premenopausal Patients With a History of Breast Cancer

**DOI:** 10.1097/og9.0000000000000172

**Published:** 2026-05-21

**Authors:** Margo Nelis, Anna Chichura

**Affiliations:** Obstetrics and Gynecology Institute, Cleveland Clinic, Cleveland, Ohio; and Department of General Surgery, Brian Moran Cancer Institute, Duly Health & Care, Schaumburg, Illinois.

## Abstract

Abnormal uterine bleeding (AUB) is a common gynecologic complaint, with first-line treatment commonly involving hormonal medication. In premenopausal patients with a history of breast cancer, both the differential diagnosis and management require special consideration. This narrative review focuses on the evaluation and treatment of chronic AUB in women with a history of breast cancer. With reference to key studies and meta-analyses, we highlight controversies and current unanswered questions about patient management.

## Abstract 2

Abnormal uterine bleeding in premenopausal breast cancer survivors necessitates individualized evaluation and often avoidance of traditional hormone-based treatment options.

Abnormal uterine bleeding (AUB) affects up to 30% of premenopausal individuals and refers to changes in menstrual cycle duration, volume, frequency, or regularity.^1^ Normal menstrual cycles range from 21 to 35 days, with bleeding lasting 2–7 days and filling 2–5 pads daily.^2^ Breast cancer is the most common cancer among women, with 2.1 million cases diagnosed worldwide in 2018, of which, 645,000 were in premenopausal women.^3^ Premenopausal breast cancer is rising, with age-standardized incidence rates increasing by 29% between 1990 and 2023. Over 80% of women diagnosed before 50 years of age survive at least 5 years and require special consideration when diagnosing and treating gynecologic conditions. Many of these patients report that their gynecologic needs remain unmet.^4^

Breast cancer is classified by histology and hormone receptor status, including estrogen receptor, progesterone receptor, and human epidermal growth factor receptor 2.^5–7^ Although the majority of breast cancers in premenopausal women are hormone receptor–positive (approximately 60–65%), this proportion is lower than in postmenopausal populations, with younger patients demonstrating relatively higher rates of human epidermal growth factor receptor 2–positive disease (approximately 15–20%) and aggressive subtypes, including triple-negative breast cancer (approximately 15–25%)^8^ Tamoxifen, a selective estrogen receptor modulator, is commonly used to treat estrogen receptor–positive breast cancer, especially in premenopausal patients. Tamoxifen acts as an estrogen antagonist in breast tissue but as an agonist in the endometrium.^9^ Triple-negative breast cancer, lacking all three markers, is a more aggressive form of breast cancer that is not responsive to antiendocrine therapy.

Hormonal factors play a key role in breast cancer. Breast cancer risk increases with prolonged estrogen exposure (eg, early menarche, late menopause, nulliparity).^5,10^ Synthetic progestins—more than estrogen alone—are more strongly associated with increased risk. Bioidentical progesterone may not initiate cancer but can promote tumor growth once present.^5,11^ This review focuses on differential diagnosis for AUB and safe, effective management in this population.

## GENERAL WORKUP OF ABNORMAL UTERINE BLEEDING

According to history, targeted tests may include a pregnancy test, complete blood count, thyroid-stimulating hormone, follicle-stimulating hormone, estradiol, Pap test, and sexually transmitted disease screening. Imaging such as saline-infused sonohysterography, transvaginal ultrasound, magnetic resonance imaging, or hysteroscopy may be indicated. Patients at risk of uterine malignancy should undergo tissue sampling, with diagnostic hysteroscopy and biopsy remaining the gold standard.

## DIFFERENTIAL DIAGNOSIS AND SPECIAL CONSIDERATIONS FOR BREAST CANCER SURVIVORS

Assessment of a patient with AUB focuses on identifying the underlying cause. The American College of Obstetricians and Gynecologists (ACOG) classifies AUB into structural and nonstructural causes using the PALM-COEIN (polyp; adenomyosis; leiomyoma; malignancy and hyperplasia; coagulopathy; ovulatory dysfunction; endometrial; iatrogenic; and not yet classified) system (Table 1).^12^ This section explores the differential diagnosis for AUB in the context of a patient with a history of breast cancer.

**Table 1.** Differential Diagnosis and Treatment of Abnormal Uterine Bleeding in Patients With a History of Breast Cancer

Pathology	Differential Diagnosis	Treatment
Polyp	Increased risk from tamoxifen use	Hysteroscopy with polypectomy NSAIDs TXA*
Adenomyosis	Increased risk from tamoxifen use	NSAIDs TXA* Hysterectomy
Leiomyoma	Increased risk from tamoxifen use	NSAIDs TXA* Radiofrequency ablation Hysteroscopy with myomectomy Laparoscopic myomectomy Uterine artery embolization Hysterectomy
Malignancy	Uterine malignancy: Germline variants in *PTEN*, *MSH2*, or *TP53* Metastatic breast cancer Estrogen-producing ovarian tumors	Treat underlying cancer
Coagulopathy	Anticoagulation therapy as treatment for VTE	Explore options for AC adjustment
Ovulatory dysfunction		Oophorectomy Ovarian function suppression (leuprolide, goserelin, triptorelin)
Iatrogenic	Ovarian function suppression Chemotherapy Tamoxifen	Consult prescribing physician to assess ability to discontinue or change medication

NSAID, nonsteroidal anti-inflammatory drug; TXA, tranexamic acid; VTE, venous thromboembolism; AC, anticoagulation.

* If not increased VTE risk. TXA may be considered only in patients without increased VTE risk.

## STRUCTURAL CAUSES

### Polyps

Endometrial polyps, soft benign growths in the uterine lining, are a common cause of AUB.^12^ Although their exact cause is unclear, polyps often express high levels of estrogen receptor and progesterone receptor, suggesting hormonal influence. Tamoxifen is known to significantly increase the risk of endometrial polyps (hazard ratio 3.90, 95% CI, 3.65–4.16), especially in patients with a history of polyps.^13,14^ In addition, polyps developed in 17.6% of patients with polyps already present before medication use compared with 12.9% in the group without any history of polyps.

### Adenomyosis

Adenomyosis, the presence of endometrial tissue within the myometrium, is another structural cause of AUB.^15^ Tamoxifen use has also been linked to increased rates of adenomyosis. In one study, adenomyosis was histologically diagnosed in 53.6% tamoxifen-treated patients compared with 18.2% of patients not on tamoxifen.^16,17^ Adenosis may be suspected based on ultrasound findings, but diagnosis can be confirmed only through histopathology after hysterectomy.^18^

### Leiomyomas

Leiomyomas (fibroids) are benign uterine tumors that are a common cause of AUB.^12^ Incidence rates of leiomyomas in premenopausal women are increased in those receiving tamoxifen compared with placebo (risk ratio 1.3, 95% CI, 1.14–1.55).^13^ Multiple case reports demonstrate rapid fibroid enlargement in premenopausal women after starting tamoxifen, possibly because of its proestrogenic effects in the uterus.^19–23^ These cases suggest that tamoxifen may promote fibroid growth, contributing to AUB, similar to its effects on polyps and adenomyosis.

### Malignancy

For a patient with a history of breast cancer, malignancy is important to rule out as a part of the workup for AUB. It is essential to explore a patient's personal and family history of germline pathogenic variants because several hereditary syndromes are associated with an elevated risk of malignancies in both the breast and uterus. These include Cowden syndrome, Lynch syndrome–associated genes, and Li-Fraumeni syndrome.^3,24^

Cowden syndrome, caused by mutation in *PTEN*, increase the lifetime risk of breast cancer to 85% and endometrial cancer to 28%.^25^ Lynch syndrome, caused by pathogenic variants in mismatch repair genes *MLH1*, *MSH2*, *MSH6*, *PMS2*, and *EPCAM*, increases endometrial cancer risk to 13.4%. The *PMS2* variant has been specifically linked to higher breast cancer risk.^26,27^ *TP53* pathogenic variants, associated with Li-Fraumeni syndrome, predispose individuals to early breast cancer and uterine carcinosarcoma.^28,29^

Although rare, metastatic breast cancer should also be considered. Although breast cancer typically spreads to the bones, lung, or liver, uterine metastases can occur, most commonly invasive lobular carcinoma.^30^ Ovarian pathologies such as hormone-secreting tumors should also be considered. Granulosa cell tumors, a type of malignant sex cord–stromal tumor, secrete estrogen and other hormones that affect hormone-sensitive tissues, leading to increased rates of breast and uterine cancers.^31^ Although family history may increase risk, no specific pathogenic variants are linked to germ cell or stromal tumors. If suspected, evaluation should include transvaginal ultrasound and laboratory tests for hormone levels and tumor markers, including inhibin A and B.^31–33^

Tamoxifen is commonly used for 5–10 years to prevent breast cancer recurrence in premenopausal women with low-risk disease.^34^ Over 50% of women on tamoxifen experience AUB.^1^ The ACOG has reported that hormonal therapy in premenopausal women is not associated with an increased risk of cancer. However, more recent population-based studies from South Korea and Taiwan have demonstrated a statistically significant increase in cancer risk within this population, highlighting ongoing uncertainty and the need for further investigation.^35–37^ However, no routine endometrial sampling is recommended in a premenopausal patients on tamoxifen; however, sampling is suggested in patients with prolonged amenorrhea or AUB.^38^

### Coagulopathy

Coagulopathies causing AUB may be inherited (eg, von Willebrand disease) or acquired. In breast cancer survivors, it is essential to consider any history of anticoagulant use. Individuals with breast cancer have a fourfold higher risk of venous thromboembolism (VTE) compared with age- and sex-matched controls without cancer. Patients with breast cancer have a 6% risk of VTE annually while undergoing chemotherapy and in the month after treatment. In addition, patients using tamoxifen have a 6% annual risk of VTE, 4 times higher than the risk before starting therapy.^39^ Clarifying whether bleeding symptoms predated cancer treatment is key because some therapies can mimic bleeding disorders.^40^

### Ovulatory Dysfunction

Ovulatory dysfunction is a key cause of AUB, ranging from amenorrhea to heavy, irregular bleeding.^12^ Many common breast cancer treatments can cause irregular cycles. This is discussed further in the iatrogenic causes of AUB.

### Endometrial

When AUB occurs in the context of regular menses and in the absence of other definable causes, the mechanism can be a primary disorder residing in the endometrium. The diagnosis of AUB-E should be determined by exclusion of other identifiable abnormalities.^40^ There are no specific considerations for AUB-E in the context of patients with breast cancer but should remain in the differential.

### Iatrogenic

It is crucial to consider iatrogenic causes of AUB in patients with breast cancer. For these patients, a history of VTE requiring anticoagulation and use of tamoxifen are common causes of AUB.

In breast cancer survivors, systemic treatments, especially chemotherapy and endocrine therapy, often impair ovarian function and lead to AUB. Evaluating cancer type, stage, hormone receptor status, age, and treatment is critical because systemic therapy affects the ovaries more than surgery or radiation.^41^

Chemotherapy can damage ovarian follicles and disrupt the ovarian environment. A 2024 review noted fibrosis, oxidative stress, immune disruption, and stem cell loss, all contributing to ovarian dysfunction.^42^ Risk of ovarian dysfunction varies by age, baseline ovarian reserve, and agents used; alkylating agents such as cyclophosphamide are especially gonadotoxic, whereas doxorubicin results in amenorrhea in 7–80% of patients.^42,43^ Immune checkpoint inhibitors may affect hormone regulation, although direct gonadotoxicity is unconfirmed. The effects of human epidermal growth factor receptor 2–targeted therapies such as trastuzumab and PD-1 inhibitors such as pembrolizumab remain unclear.^44,45^

Aromatase inhibitors block the peripheral conversion of androgens to estrogens but do not inhibit ovarian estrogen production. This medication class, once used only in postmenopausal women, has now shown to benefit premenopausal patients when paired with ovarian suppression.^46,47^ Incomplete ovarian suppression can result in AUB and is an important consideration because it can render aromatase inhibitors ineffective.

### Treatment

Abnormal uterine bleeding can typically be treated with medical or surgical management. When considering treatment options, it is important to note whether the bleeding is an acute or chronic issue. Acute AUB is defined as an episode of heavy bleeding that is of sufficient quantity to require immediate intervention to prevent further blood loss.^48,49^ This review focuses on the treatment of chronic AUB and does not specifically discuss the treatment options for an acute bleeding episode.^48,49^

### Medical Options

Abnormal uterine bleeding is often treated with medical management. The current approved options for AUB in the general population are the levonorgestrel intrauterine system (LNG-IUS), combined oral contraceptive pills, continuous oral progestins, and tranexamic acid. Nonsteroidal anti-inflammatory drugs (NSAIDs) may be used with hormonal methods and tranexamic acid to decrease menstrual bleeding.^50^

## HORMONAL OPTIONS

### Use of the Levonorgestrel Intrauterine System

Progestin-containing intrauterine systems such as LNG-IUS are commonly used to treat AUB.^51^ Although the LNG-IUS acts locally, it still produces measurable systemic hormone levels of levonorgestrel.^38^ Breast cancer is listed as a contraindication in the labeling for LNG-IUS, regardless of receptor status. The Centers for Disease Control and Prevention classifies the LNG-IUS as category 4 (unacceptable risk) for women with current breast cancer and category 3 (risks usually outweigh benefits) for those diagnosed more than 5 years ago.^52^ The ACOG also recommends nonhormonal methods for breast cancer survivors.^53^ If in situ at time of breast cancer diagnosis, patients should be counseled on removal and nonhormonal alternatives. Limited evidence suggests that LNG-IUS may help with tamoxifen-related endometrial changes and could be considered for select hormone receptor–negative patients.^54,55^ A 2021 study found no significant difference in breast cancer recurrence in both hormone and nonhormonal tumors between hormonal contraceptive users, including progesterone IUS, and nonusers.^56^ Similarly, a 2008 case–control study examined premenopausal patients with hormone-positive and hormone-negative breast cancer and fond no significant difference in recurrence rates between LNG-IUS users and controls but did note fewer polyps in the LNG-IUS group.^57^ In addition, another study found no increase in recurrence or breast cancer–related deaths with LNG-IUS use in patients on tamoxifen, although the results were not statistically significant.^58^ Despite these studies, LNG-IUS is not recommended for routine use in patients with breast cancer given the number of alternatives available. In refractory cases, LNG-IUS should be considered only after consultation with the patient's medical oncologist.

### Use of Hormonal Oral Contraceptive Pill

Hormonal treatments for AUB, including combined oral contraceptive pills and progestins, improve cycle control and reduce bleeding but are generally not recommended for breast cancer survivors because of hormonal exposure.^59^

Systemic menopausal hormone therapy increases recurrence risk in hormone receptor-positive breast cancer (hazard ratio 1.80, 95% CI, 1.15–2.82, *P*=.010).^60–63^ Although data on estrogen receptor–negative and triple-negative breast cancer remain limited, hormone use in triple-negative breast cancer is controversial and best avoided. Risk-reducing oophorectomy decreases breast cancer risk in *BRCA1/2* carriers, especially if performed before 40 years of age, highlighting the role of hormones in both estrogen receptor–positive and estrogen receptor–negative disease.^64^

Although many studies link hormonal contraception to increased breast cancer risk, few address recurrence.^10,53,65–67^ One study found no difference in recurrence rates between users and nonusers, whereas others report a modest increase, especially with early or prolonged use (hazard ratio 1.20, 95% CI, 1.14–1.26).^10,68,69^

Progestins vary by generation and suppress the hypothalamic–pituitary–ovarian axis, but their safety in breast cancer remains unclear.^4^ Animal models suggest that progestins may promote tumor growth and metastasis. Some studies associate progestin-only contraceptives with higher breast cancer risk (incidence risk ratio 1.32), particularly in the first 5 years of use, whereas combined contraceptives show a smaller risk (incidence risk ratio 1.03) that normalizes after 10 years.^70^ Although agents such as medroxyprogesterone acetate may have antitumor effects in select settings, the overall evidence supports avoiding progestins for AUB in breast cancer survivors.^71,72^

Systemic hormones should be used with extreme caution in this population and may be considered only after nonhormonal options are exhausted and in consultation with the patient's oncologist.

## NONHORMONAL OPTIONS

### Nonsteroidal Anti-inflammatory Drug Agents

Nonsteroidal anti-inflammatory drugs are a nonhormonal option for managing AUB by reducing elevated prostaglandin levels and addressing dysmenorrhea. Nonsteroidal anti-inflammatory drugs may reduce bleeding by 25–35% in up to 75% of women with heavy menstrual bleeding. Side effects include gastrointestinal, renal, cardiovascular, and hematologic risks.^4,48,49,64^

Overall, we advocate for the use of NSAIDs for patients with AUB given their demonstrated efficacy and favorable safety profile if there are no contraindications.

### Tranexamic Acid

Tranexamic acid is approved by the U.S. Food and Drug Administration for heavy menstrual bleeding and works by stabilizing clots.^73^ Abnormal uterine bleeding can be managed with 1,300 mg orally three times daily for 5 days, with the option to repeat monthly with menstruation.^74^

Tranexamic acid increases the risk of VTE, a risk that is already elevated in breast cancer patients. One study found that tranexamic acid use tripled VTE risk (adjusted odds ratio 3.3, 95% CI, 1.3–9.1, *P*=.02) in this population.^75^ Risk factors for VTE include increased age, obesity, cancer stage, treatment type, and tamoxifen use (especially in the first 6 months of use).^76^

Although tranexamic acid has shown benefits in reducing postoperative bleeding without increasing VTE during breast cancer surgery, prolonged use should be avoided in patients at high risk.^77,78^ Short-term use may be appropriate for individuals at low risk. We recommend avoiding use in combination with tamoxifen.

## OVARIAN SUPPRESSION

Ovarian suppression for AUB can be achieved medically or surgically. Both gonadotropin-releasing hormone (GnRH) agonists and antagonists work to suppress sex hormone production. Approved by the U.S. Food and Drug Administration for AUB resulting from fibroids, GnRH antagonists have been shown to significantly improve AUB symptoms.^79^ The GnRH agonists such as leuprolide, goserelin, and triptorelin can be used to treat AUB, fibroids, and endometriosis and are commonly used as a component of breast cancer treatment.^80^ Leuprolide has been shown to significantly reduce transfusion needs in patients with hematologic malignancies and AUB.^81^ We recommend leuprolide 11.25 mg intramuscularly as a first-line treatment for AUB in patients with breast cancer.^74^ According to leuprolide prescribing information, this formulation provides sustained bioavailability for 2 months and may be administered at appropriate 2-month intervals. Side effects include hot flashes, breast tenderness, decreased bone mineral density, and nausea.^74,82^ Although the use of add-back therapy is often recommended for patients receiving GnRH antagonists to reduce the risk of osteoporosis, we do not recommend add-back therapy in this patient population.^83^

The 2015 SOFT/TEXT trial (Suppression of Ovarian Function Trial/Tamoxifen and Exemestane Trial) found no significant benefit in disease-free survival when ovarian suppression, triptorelin, oophorectomy, or ovarian irradiation was added to tamoxifen in premenopausal patients. A substudy of women on ovarian function suppression and aromatase inhibitors showed that over 17% had breakthrough elevation in E2 levels, most notably in those younger than 35 years of age, those with higher body mass index (BMI), or those who had not received chemotherapy.^84^ This finding highlights the limited and inconsistent efficacy of ovarian suppression while emphasizing the importance of considering it as a cause of AUB.

Surgical suppression such as oophorectomy may be considered for refractory AUB when estrogen is a contributing factor.^85^ Oophorectomy may be appropriate for premenopausal patients with breast cancer requiring long-term suppression such as in metastatic disease or in patients with a genetic mutation predisposing to ovarian cancer.^86^ However, routine oophorectomy for AUB without clear pathology is discouraged because of risks such as early death (hazard ratio 1.67, 95% CI, 1.16–2.40, *P*=.006), cardiovascular disease, cognitive decline, osteoporosis, and decreased sexual function, particularly in patients younger than 45 years of age.^87,88^ When oophorectomy is indicated, a minimally invasive approach—most commonly laparoscopic bilateral salpingo-oophorectomy—remains the standard of care because of its lower morbidity and faster recovery. The surgical technique should be tailored to the underlying indication,^89,90^ These recommendations apply to AUB; in other cases such as in patients with pathogenic germline variants, oophorectomy may offer survival benefit.

### Surgical Options

Abnormal uterine bleeding caused by uterine or cervical abnormalities, independently of a history of breast cancer, can commonly be treated surgically.^1^ The surgical approach should be customized for each patient by evaluating the underlying structural cause of the bleeding while considering fertility plans, medical comorbidities, age, projected duration of treatment, potential need for future cavitary assessment, and the patient's preference.^8^ Options include hysteroscopy with polypectomy, myomectomy (either through hysteroscopy or surgically), endometrial ablation, uterine artery embolization, oophorectomy, ovarian ablation, or hysterectomy.^91,92^

## CONCLUSION

It is essential to consider the unique aspects of the PALM-COEIN classification and treatment options when managing AUB in premenopausal breast cancer survivors.

In the differential diagnosis, special attention should be given to the association between tamoxifen use and structural causes of AUB. In addition, the risk of cancer recurrence and the presence of genetic mutations that predispose individuals to both breast and uterine cancers must be evaluated.

Hormonal therapies are generally considered unsafe in this population. Instead, nonhormonal options such as tranexamic acid, NSAIDs, and leuprolide are preferred.

Although the discussion is grounded in current literature, all treatment decisions should be made in collaboration with the patient's oncologist to ensure a comprehensive, individualized approach.
